# An open-label study evaluating the safety and efficacy of budesonide in patients with IgA nephropathy at high risk of progression

**DOI:** 10.1038/s41598-023-47393-1

**Published:** 2023-11-17

**Authors:** Bogdan Obrișcă, Alexandra Vornicu, Valentin Mocanu, George Dimofte, Andreea Andronesi, Raluca Bobeică, Roxana Jurubiță, Bogdan Sorohan, Nicu Caceaune, Gener Ismail

**Affiliations:** 1 Department of Nephrology, “Carol Davila” University of Medicine and Pharmacy, Bucharest, Romania; 2 Department of Nephrology, Fundeni Clinical Institute, Bucharest, Romania; 3 Department of Internal Medicine, Fundeni Clinical Institute, Bucharest, Romania

**Keywords:** IgA nephropathy, Chronic kidney disease

## Abstract

We sought to evaluate the efficacy and safety of budesonide (Budenofalk) in the treatment of patients with IgA Nephropathy. We conducted a prospective, interventional, open-label, single-arm, non-randomized study that enrolled 32 patients with IgAN at high risk of progression (BUDIGAN study, ISRCTN47722295, date of registration 14/02/2020). Patients were treated with Budesonide at a dose of 9 mg/day for 12 months, subsequently tapered to 3 mg/day for another 12 months. The primary endpoints were change of eGFR and proteinuria at 12, 24 and 36 months. The study cohort had a mean eGFR and 24-h proteinuria of 59 ± 24 ml/min/1.73m^2^ and 1.89 ± 1.5 g/day, respectively. Treatment with budesonide determined a reduction in proteinuria at 12-, 24- and 36-months by -32.9% (95% CI − 53.6 to − 12.2), − 49.7% (95% CI − 70.1 to − 29.4) and − 68.1% (95% CI − 80.6 to − 55.7). Budesonide determined an eGFR preservation corresponding to a 12-, 24- and 36-months change of + 7.68% (95% CI − 4.7 to 20.1), + 7.42% (95% CI − 7.23 to 22.1) and + 4.74% (95%CI − 13.5 to 23), respectively. The overall eGFR change/year was + 0.83 ml/min/y (95% CI − 0.54 to 4.46). Budesonide was well-tolerated, and treatment emergent adverse events were mostly mild in severity and reversible. Budesonide was effective in the treatment of patients with IgAN at high-risk of progression in terms of reducing proteinuria and preserving renal function over 36 months of therapy.

## Introduction

Immunoglobulin A nephropathy (IgAN) is the most common primary glomerular disease worldwide^1, 2^. Up to 50% of patients will progress to end-stage renal disease (ESRD) within 20 years from diagnosis^1, 3^. Nonetheless, a recent report from a UK Registry that enrolled 2439 patients with IgAN showed that almost all patients were at risk of progression to kidney failure within their expected lifetime unless an eGFR rate loss below 1 ml/min/year is achieved^3^. Moreover, the current accepted threshold for persistent proteinuria to define a high risk of progression (0.75–1 g/day) may be debated as 30% and 20% of those with time-averaged proteinuria of 0.44–0.88 g/g creatinine and less than 0.44 g/g, respectively, developed kidney failure within 10 years^3–5^.

The efficacy of immunosuppression in the management of IgAN remains highly controversial^1^. The STOP-IgAN concluded that adding immunosuppression on top of optimized supportive care was not associated with improved renal outcomes, while significantly increasing the infection risk^6, 7^. Subsequently, the initial TESTING trial showed a significant 63% reduction in the risk of the primary renal outcome, but the study was prematurely discontinued due to an excess of adverse events (mainly infections) associated with methylprednisolone use^8^. The excess of adverse events was reduced, but not eliminated, following a dose reduction of methylprednisolone to 0.4 mg/kg/d and addition of antibiotic prophylaxis, while maintaining a similar efficacy in terms of reducing the risk of the primary renal outcome^9^. Nevertheless, the effect of a 6–8-month course of methylprednisolone on proteinuria reduction was lost after 3 years, while the annual rate of loss of kidney function remained substantially high (− 2.5 ml/min/y), despite being lower than the placebo group (− 4.97 ml/min/y)^9^. While efficient at mitigating the glomerular inflammation, systemic corticosteroids do not target the initial pathogenic events of IgAN and this might explain their limited efficacy in improving long-term renal outcomes in this disease^10^.

The gut-kidney axis hypothesis has been supported by several studies over the past decades to underlie the pathogenesis of IgAN^11, 12^. The earliest pathogenic event in IgAN involves the B-lymphocytes from Peyer’s patches that are primed to produce galactose-deficient molecules of IgA1 (Gd-IgA1) in response to microbial or dietary antigens^1^. This has led to the development of a targeted-released formulation of budesonide (Nefecon) designed to release the active drug in the distal ileum and proximal part of ascending colon, sites with the highest density of Peyer’s patches^1, 13^. In the NEFIGAN and NefIgArd trial, treatment with Nefecon for 9 months led to a significant reduction in proteinuria and stabilization of kidney function^14–16^. Nonetheless, the selective mechanism of action of Nefecon remains to be proven^10^, while other budesonide formulations that are prescribed for the treatment of inflammatory bowel disease (IBD) may have a significant anti-inflammatory effect on the gut-associated lymphoid tissue^17, 18^. In a previous retrospective study, we have shown that a 24-month budesonide (Budenofalk) treatment was associated with a significant reduction in proteinuria and preservation of renal function^19^.

Accordingly, we have conducted a prospective, non-randomized study to evaluate the safety and efficacy of budesonide (Budenofalk) treatment in patients with IgAN at high risk of progression.

## Material and methods

### Study design and population

The BUDIGAN study (ISRCTN47722295, date of registration 14/02/2020) is a prospective, interventional, open-label, non-randomized study that enrolled 32 patients with IgAN, at high risk of progression. The study design is in line with our previous report^19^.

The inclusion criteria were: age ≥ 18 years, patients with a histological diagnosis of primary IgAN, high risk of progression defined as persistent proteinuria over 1 g/day despite adequate renin–angiotensin–aldosterone system (RAAS) blockade or patients with proteinuria between 0.5 and 1 g/day after RAAS blockade if they had additional risk factors for progression (estimated glomerular filtration rate below 60 ml/min/1.73m^2^, presence of proliferative lesions on kidney biopsy). We excluded patients with: age under 18 years, those with IgAN associated with other disorders (viral infections, autoimmune disorders, malignancy) or Henoch-Schönlein purpura, those with an eGFR below 20 ml/min/1.73m^2^, nephrotic syndrome or a rapidly progressive clinical course, patients with proteinuria below 0.5 g/day after adequate RAAS blockade, those with severe histological lesions of activity or chronicity (endocapillary hypercellularity in over 50% of examined glomeruli, crescents in over 30% of examined glomeruli, presence of fibrinoid necrosis, global glomerulosclerosis in over 50% of examined glomeruli), patients with diabetes mellitus or active infections, patients that received prior immunosuppression.

The study was conducted after institutional approval (The Ethics Council of Fundeni Clinical Institute, Registration number: 1975, 14th January 2020). All methods were conducted in compliance with the Declaration of Helsinki and the relevant guidelines and regulations. Informed consent was obtained from all participants or, if participants are under 16, from a parent and/or legal guardian.

### Treatment

All patients had received a stable dose of an angiotensin converting enzyme (ACE) inhibitor or angiotensin receptor blocker (ARB) therapy for a period of at least 3 months prior to budesonide initiation. The dose of ACEI/ARB was titrated to a maximum tolerated dose in order to achieve a target blood pressure (BP) of 125/75 mmHg or less and a proteinuria level below 0.5 g/day. In addition, the patients were advised on salt restriction, smoking and nonsteroidal anti-inflammatory agent avoidance.

Patients that after RAAS blockade initiation were considered to be at high-risk of progression were started on budesonide at a dose of 9 mg/day, for the first 12 months, followed by a subsequent dose reduction to 3 mg/day for the next 12 months. Budesonide (Budenofalk) is a controlled-release, gastro-resistant, pH-modified oral steroid formulation^17, 18^. It has a maximum release in the distal ileum and proximal colon and extensive first-pass metabolism, being associated only with mild and transient reductions in plasma cortisol levels and minimal steroid-related side effects^17, 18^.

### Study follow-up and data collection

The study follow-up period was 36 months. At baseline the following data were collected: age, gender, mean arterial pressure, renal function assessed by serum creatinine and eGFR (estimated glomerular filtration rate calculated by the 2009 CKD-EPI equation^20^), serum uric acid, serum albumin, 24-h proteinuria (g/d) and hematuria (cells/μL). Only patients with a histological diagnosis of primary IgAN were considered for study inclusion. The diagnosis of IgAN was based on light microscopy, immunofluorescence (dominant or codominant IgA in the mesangium) and electron microscopy (para-mesangial electron-dense deposits). All kidney biopsies were reviewed and scored according to the 2016 Oxford Classification^21^. In addition to the MESTC score, the histological assessment included the percentage of glomeruli with global sclerosis. All patients underwent a systematic screening for disorders reported to be associated with IgAN^22^.

### Study outcomes

The efficacy study outcomes were the effects of Budenofalk on proteinuria and eGFR at 12, 24 and 36 months. The safety outcomes were treatment emergent adverse events.

### Statistical analysis

Continuous variables were expressed as either mean (± standard deviation or 95% confidence interval) or median (interquartile range, IQR: 25th–75th percentiles) and categorical variables as percentages. Differences between groups were assessed in case of continuous variables by Student *t* test, Mann–Whitney test, one-way ANOVA or Kruskal–Wallis test, according to the distribution of dependent variables and the level of independent variable, and in case of categorical variables by χ^2^ test or Fisher’s exact test. In all analyses, p values are two-tailed and all p values less than 0.05 were considered statistically significant.

Statistical analyses were performed using the SPSS program (SPSS version 20, Chicago, IL), and GraphPad Prism version 9.3.1 (1992–2021 GraphPad Software, LLC).

## Results

### Study population

**Figure 1:**
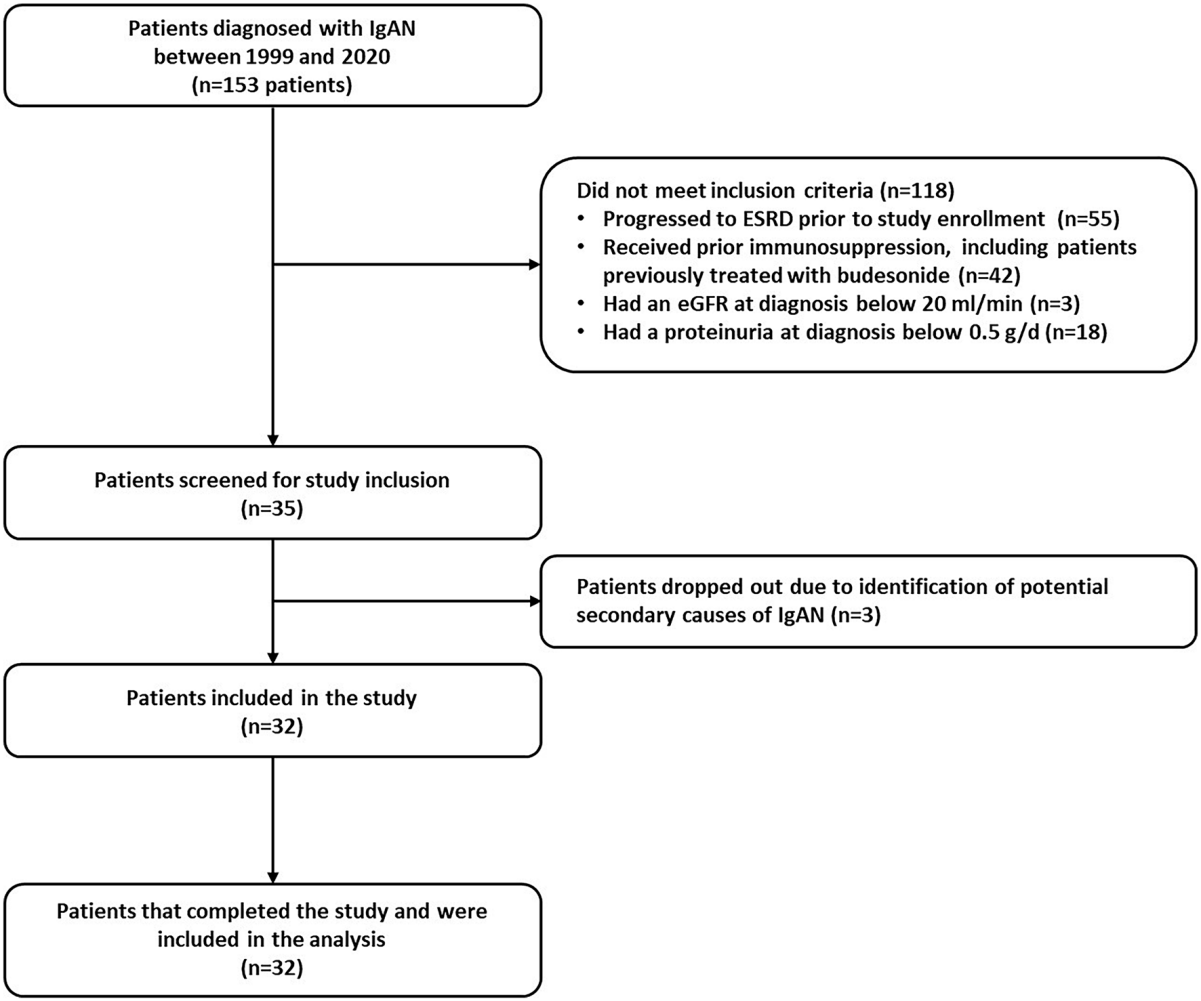
Study flow-chart.

Between 1999 and 2020, a total of 153 patients were diagnosed with IgAN in our department, of whom 118 did not meet the inclusion criteria (Fig. 1). A total of 35 patients were considered for study inclusion. Three patients were dropped out of the study (were further identified to have potential secondary causes of IgAN), leaving a final cohort of 32 patients. The baseline characteristics of the study cohort are depicted in Table 1. The study population had a mean age at treatment initiation of 41.7 ± 9.4 years, the majority being males (71.9%), while the mean time from IgAN diagnosis to study inclusion was 1.3 ± 2.3 years. The mean serum creatinine and eGFR were 1.61 ± 0.67 mg/dl and 59 ± 24 ml/min/1.73m^2^, respectively, with 46.9% of patients having an eGFR below 60 ml/min/1.73m^2^. The mean 24-h proteinuria was 1.89 ± 1.5 g/d, while 87.5% of patients had microscopic hematuria. All patients had a 24-h proteinuria over 0.75 g/day, with thirty-seven percent of the study cohort having a 24-h proteinuria over 2 g/d, while 4 patients (12.5%) had a 24-h proteinuria over 3.5 g/d. The patients with nephrotic-range proteinuria at baseline did not have a concomitant reduction in serum albumin levels and did not fulfil the criteria for nephrotic syndrome.

**Table 1:** Baseline characteristics of the study cohort.

Variable	Value
Number of pts	32
Gender (%m)	71.9%
Age (y)	41.7 ± 9.4
MAP (mmHg)	93 ± 13
Serum Creatinine (mg/dl)	1.61 ± 0.67
eGFR (ml/min/1.73m^2^)	59 ± 24
CKD stage (%)	
G1	12.5%
G2	40.6%
G3	31.3%
G4	15.6%
Uric acid (mg/dl)	6.9 ± 1.7
Serum albumin (g/dl)	4.2 ± 0.3
24-h proteinuria (g/day)	1.89 ± 1.5
Proteinuria level (%)	
0.75–2 g/day	62.5%
2–3.5 g/day	25%
> 3.5 g/day	12.5%
Hematuria (cells/μL )	42 (IQR:25–93)
Patients with microhematuria (%)	87.5%
Conservative treatment	
Use of any RAAS blockade (% of patients)	
ACE inhibitor alone	71.9%
ARB alone	28.1%
Both ACE inhibitor and ARB	0%
Level of RAAS blockade as a percentage of maximum allowable dose	
< 50%	31.3%
≥ 50%	68.7%
Use of statins (% of patients)	31.2%
Biopsy features	
Percentage of glomeruli with global sclerosis (%)	13.4% (IQR:0–28.2)
MESTC score (% of pts.)	
M1	84.4%
E1	21.9%
S1	75%
T1/T2	21.9%/12.5%
C1/C2	9.4%/9.4%

*pts* patients, *m* males, *y* years, *MAP* mean arterial pressure, *eGFR* estimated glomerular filtration rate, *CKD* chronic kidney disease, *M* mesangial hypercellularity, *E* endocapillary hypercellularity, *S* segmental sclerosis, *T* tubular atrophy/interstitial fibrosis, *C* crescents, *RAAS* renin–angiotensin–aldosterone system, *ACE* angiotensin-converting enzyme, *ARB* angiotensin II type 1 receptor blocker.

The mean arterial pressure at baseline was 93 ± 13 mmHg, while the median number of antihypertensive agents used was 1 (IQR: 1–2). As per study protocol, all patients were on an RAAS blocker, the majority of patients being on ACE inhibitors (71.9%). In almost 70% of the study cohort the doses of RAAS blockers were over 50% of the maximum allowable dose (Table 1). In addition, 31.2% of patients did also receive statin therapy.

In terms of histological findings, the median percentage of glomeruli with global sclerosis was 13.4% (IQR: 0–28.2). In addition, 84.4% of patients had mesangial hypercellularity, 21.9% had endocapillary hypercellularity, 75% had segmental sclerosis and 34.4% had at least 25% of the cortical area with tubular atrophy and interstitial fibrosis. Crescents were present in at least one glomerulus in 18.8% of patients.

### Proteinuria and renal function evolution

**Figure 2:**
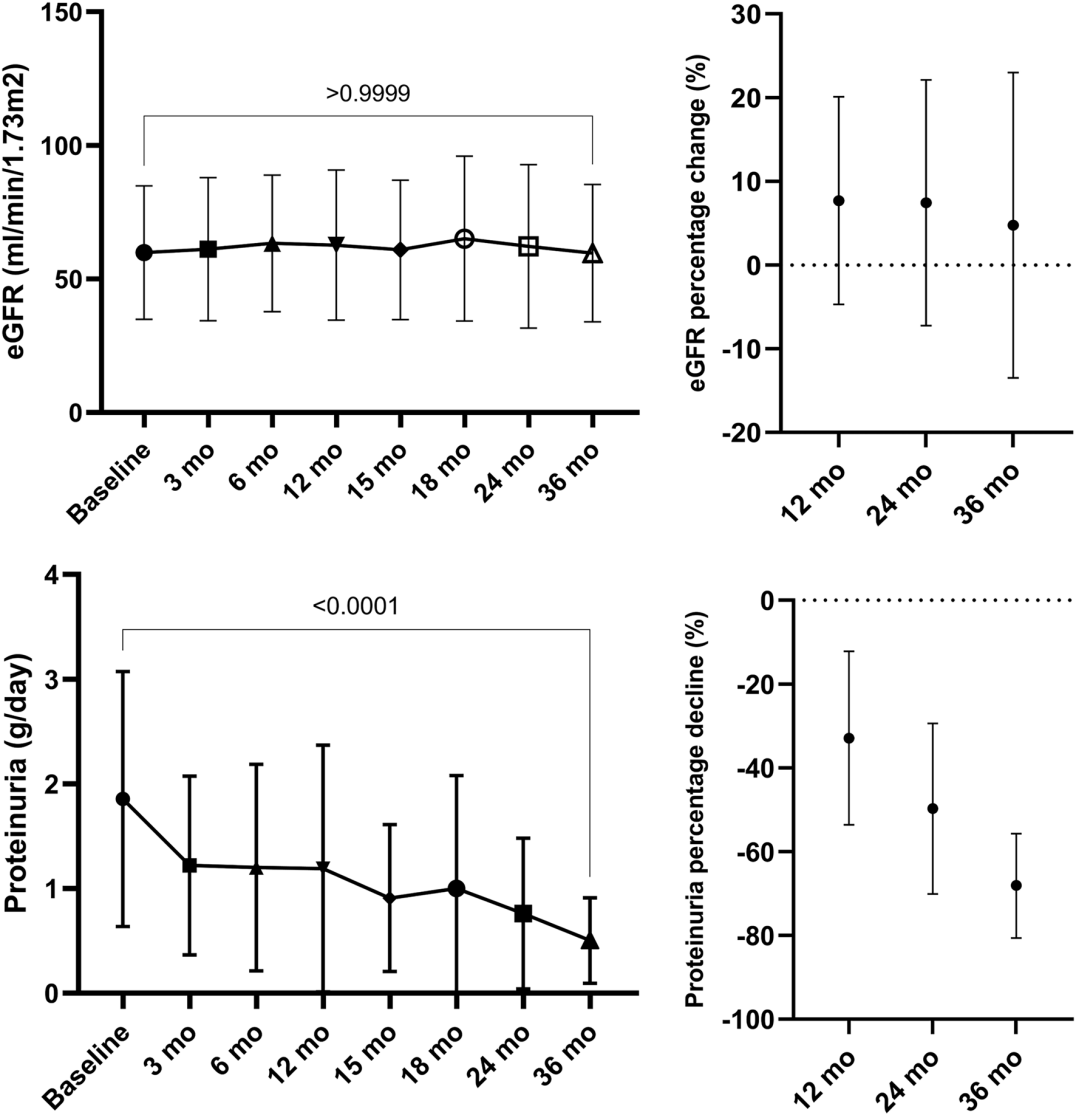
eGFR and proteinuria evolution.

Treatment with budesonide was associated with a significant decline in proteinuria and stabilization of eGFR over the 36 months of the study (24 months of treatment and 12 months of post-treatment follow-up) (Fig. 2). All patients included in the study completed the 36 months of follow-up period.

**Figure 3:**
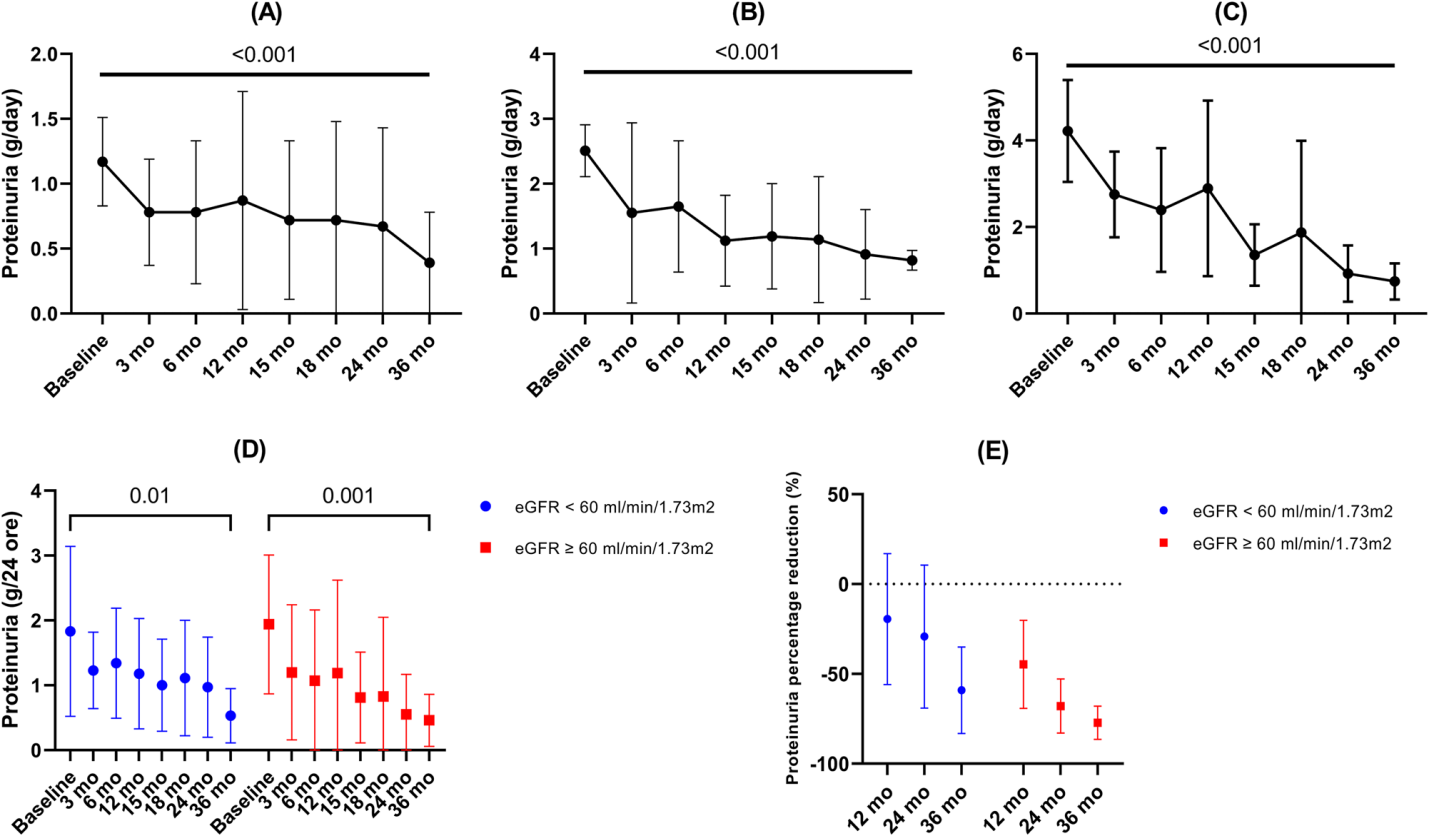
Proteinuria evolution according to study subgroups. (**A**) Patients with 24-h proteinuria < 2 g/d; (**B**) Patients with 24-h proteinuria 2–3.5 g/d; (**C**) Patients with 24-h proteinuria > 3.5 g/d; (**D**) Proteinuria evolution according to baseline eGFR; (**E**) Percentage reduction of proteinuria according to baseline eGFR.

Proteinuria significantly decreased from 1.89 ± 1.5 g/d at baseline to 0.5 ± 0.4 g/d at 36 months (p < 0.001) (Fig. 2). The corresponding mean percentage reduction in proteinuria at 12, 24 and 36 months were − 32.9% (95% CI − 53.6 to − 12.2), -49.7% (95% CI − 70.1 to − 29.4) and − 68.1% (95% CI − 80.6 to − 55.7), respectively. In addition, during the 12 months of post-treatment follow-up, there was a further decline in proteinuria level. Treatment with budesonide was associated with a significant decline in proteinuria irrespective of baseline proteinuria (Table 2, Fig. 3A–C). Baseline proteinuria was similar in patients with eGFR below or over 60 ml/min/1.73m^2^ (p = 0.81). Following treatment with budesonide, patients with a baseline eGFR over 60 ml/min/1.73m^2^ had a tendency for a greater decline of proteinuria at 12, 24 and 36 months (p = 0.23, p = 0.06 and p = 0.11, respectively) compared to those with a baseline eGFR below 60 ml/min/1.73m^2^ (Table 2, Fig. 3D–E). At the end of follow-up period 75% of patients had a proteinuria below 0.75 g/d, while 4 patients showed either a minimal decrease (less than 15%) or an increase in the proteinuria level throughout the study.

**Table 2:** Proteinuria evolution.

Period	Entire cohort (± SD)	Baseline proteinuria < 2 g/d (± SD)	Baseline proteinuria 2–3.5 g/d (± SD)	Baseline proteinuria > 3.5 g/d (± SD)	Baseline eGFR < 60ml/min (± SD)	Baseline eGFR ≥ 60ml/min (± SD)
Baseline	1.89 ± 1.5	1.17 ± 0.34	2.51 ± 0.4	4.22 ± 1.18	1.83 ± 1.31	1.94 ± 1.07
3 mo	1.22 ± 0.85	0.78 ± 0.41	1.55 ± 1.39	2.75 ± 0.99	1.23 ± 0.59	1.2 ± 1.03
6 mo	1.2 ± 0.98	0.78 ± 0.55	1.65 ± 1.01	2.39 ± 1.43	1.34 ± 0.85	1.07 ± 1.09
12 mo	1.19 ± 1.17	0.87 ± 0.84	1.12 ± 0.7	2.89 ± 2.03	1.18 ± 0.85	1.19 ± 1.43
15 mo	0.9 ± 0.7	0.72 ± 0.61	1.19 ± 0.81	1.35 ± 0.71	1 ± 0.71	0.81 ± 0.7
18 mo	0.96 ± 1.07	0.72 ± 0.76	1.14 ± 0.97	1.87 ± 2.12	1.11 ± 0.89	0.83 ± 1.22
24 mo	0.76 ± 0.72	0.67 ± 0.76	0.91 ± 0.69	0.92 ± 0.65	0.97 ± 0.77	0.55 ± 0.62
36 mo	0.5 ± 0.4	0.39 ± 0.39	0.82 ± 0.15	0.74 ± 0.42	0.53 ± 0.42	0.46 ± 0.4
Percentage reduction (95%CI)						
12 mo	− 32.9% (− 53.6, − 12.2)	− 26.4% (− 57, 4.2)	− 54.7% (− 77.8, − 31.5)	− 22.1% (− 100, − 72.6)	− 19.5% (− 56, 16.9)	− 44.7% (− 69.2, − 20.2)
24 mo	− 49.7% (− 70.1, − 29.4)	− 42.2% (− 73.5, − 10.9)	− 55.4% (− 84.1, − 26.6)	− 76.1% (− 100, − 45.2)	− 29.2% (− 69.1, 10.6)	− 67.9% (− 83, − 52.8)
36 mo	− 68.1% (− 80.6, − 55.7)	− 65.6% (− 83.2, − 48)	− 65.2% (− 79.9, − 50.4)	− 81.2% (− 100, − 60.8)	− 59.1% (− 83.2, − 35.1)	− 77.2% (− 86.4, − 67.9)

*eGFR* estimated glomerular filtration rate, *mo* months, *SD* standard deviation, *CI* confidence interval.

**Figure 4:**
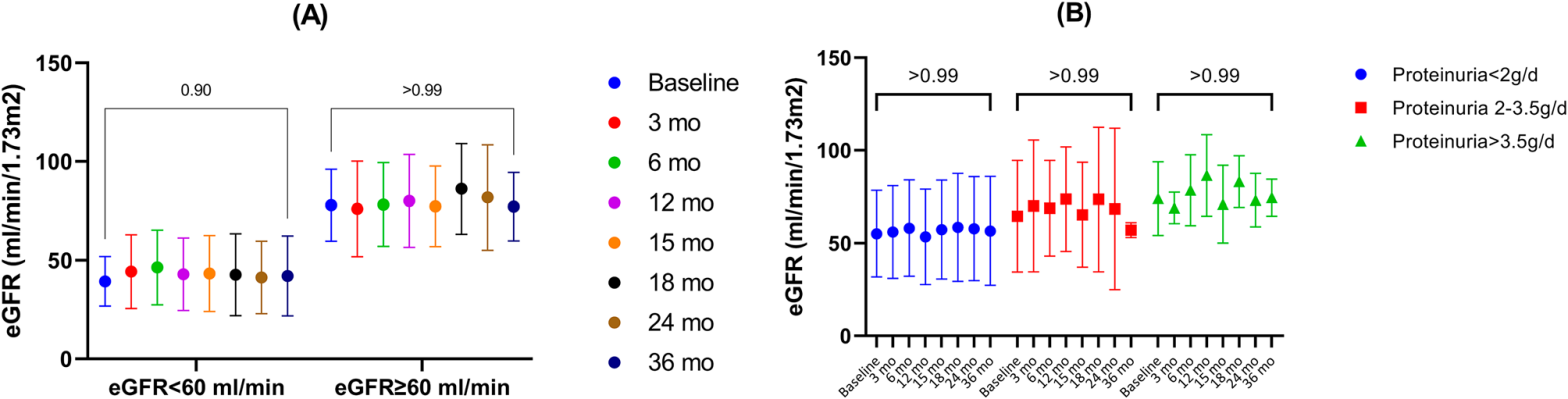
eGFR evolution. (**A**) According to baseline eGFR; (**B**) According to baseline 24-h proteinuria.

Estimated GFR showed stabilization throughout the study follow-up period, with a mean of 59 ± 24 ml/min and of 59.6 ± 25.7 ml/min at baseline and 36 months, respectively (Table 3). The corresponding mean eGFR percentage change at 12, 24 and 36 months were + 7.68% (95% CI − 4.7 to 20.1), + 7.42% (95% CI − 7.23 to 22.1) and + 4.74% (95% CI − 13.5 to 23), respectively (Table 3, Fig. 2). Despite the fact that during the treatment period the eGFR had a tendency to increase, during the 12 months of post-treatment follow-up the mean eGFR declined to the baseline value (Table 3). Overall, the eGFR change/year was + 0.83 ml/min/y (95% CI − 0.54 to 4.46), with 15.6% (n = 5) of the study cohort showing an eGFR decline over 5 ml/min/y. One patient had an eGFR below 10 ml/min/1.73m^2^ and another patient had an eGFR of 14 ml/min/1.73 m^2^ at the end of the follow-up period. However, these patients had a late diagnosis of IgAN with a baseline eGFR of 28 and 26 ml/min/1.73m^2^, respectively, and an eGFR decline of 6.6 ml/min/y and 4 ml/min/y, respectively. Nonetheless, the patient with an eGFR of less than 10 ml/min/1.73m^2^ by the end of follow-up showed severe histological features with a MESTC score of M1E0S1T2C2. The results were consistent when analyzing the eGFR change according to baseline proteinuria or baseline eGFR (Table 3, Fig. 4). In addition, similar to the analysis of the entire cohort, the trend for the attenuation of the treatment effect on eGFR during the 12 months of follow-up was maintained in the subgroup analysis.

**Table 3:** eGFR evolution.

Period	Entire cohort (± SD)	Baseline proteinuria < 2 g/d (± SD)	Baseline proteinuria 2–3.5 g/d (± SD)	Baseline proteinuria > 3.5 g/d (± SD)	Baseline eGFR ≥ 60ml/min (± SD)	Baseline eGFR < 60ml/min (± SD)
Baseline	59 ± 24	55.1 ± 23.3	64.5 ± 30.1	74 ± 19.8	77.8 ± 18.2	39.3 ± 12.5
3 mo	61.1 ± 26.8	56 ± 24.9	70 ± 35.5	69 ± 8.5	76 ± 24.2	44.2 ± 18.7
6 mo	63.3 ± 25.5	58.1 ± 25.9	68.8 ± 25.8	78.5 ± 19	78.2 ± 21.2	46.4 ± 18.9
12 mo	62.6 ± 28.1	53.4 ± 25.7	73.7 ± 28.1	86.5 ± 21.9	80 ± 23.4	42.9 ± 18.4
15 mo	60.8 ± 26	57.3 ± 26.6	65.3 ± 28.2	71 ± 20.9	77.3 ± 20.4	43.3 ± 19.2
18 mo	65.1 ± 30.8	58.5 ± 29.1	73.5 ± 38.9	83.2 ± 13.9	86.1 ± 22.9	42.6 ± 20.7
24 mo	62.2 ± 30.6	57.8 ± 28	68.4 ± 43.5	73.2 ± 14.4	81.7 ± 26.7	41.3 ± 18.3
36 mo	59.6 ± 25.7	56.6 ± 29.3	57 ± 4.3	74.5 ± 9.9	77.1 ± 17.3	42.1 ± 20.2
Percentage change (95%CI)						
12 mo	+ 7.68% (− 4.7, 20.1)	− 0.41% (− 16.2, 15.4)	+ 20.7% (− 6.4, 47.8)	+ 22.1% (− 39.1, 83.4)	+ 4.44 (− 10.2, 19.1)	+ 11.3 (− 11.4, 34.1)
24 mo	+ 7.42% (− 7.23, 22.1)	+ 6.75% (− 14, 27.5)	+ 11.9% (− 21.5, 45.5)	+ 2.92% (− 41.1, 47)	+ 8.06% (− 5.7, 21.8)	+ 6.7% (− 22, 35.5)
36 mo	+ 4.74% (− 13.5, 23)	+ 4.61% (− 21.4, 30.6)	− 3.23% (− 38, 50)	+ 4.1% (− 31, 39.3)	+ 3.53% (− 5.5, 12.6)	+ 5.95% (− 32.7, 44.6)

*eGFR* estimated glomerular filtration rate, *mo* months, *SD* standard deviation, *CI* confidence interval.

**Figure 5:**
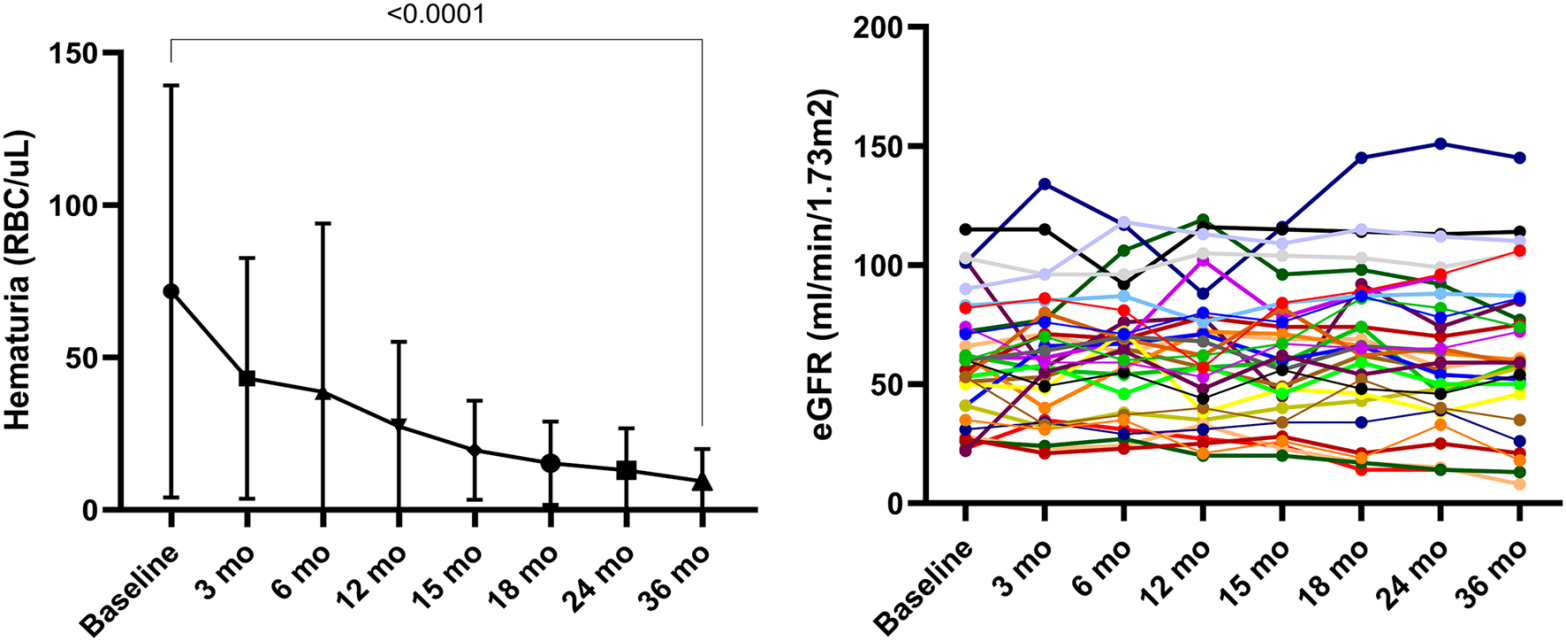
Hematuria evolution and individual eGFR evolution.

In addition to the improvement in proteinuria and eGFR stabilization, treatment with budesonide led to a significant decline in hematuria (Fig. 5). At the last follow-up, 93.7% of patients had a remission of hematuria.

### Adverse events

Overall, the 24-month treatment with budesonide was well tolerated (Table 4). All adverse events were mild or moderate in severity and did not lead to treatment discontinuation. Of the total 15 adverse events reported, 3 were gastro-intestinal symptoms (mild epigastric pain, bloating, nausea) judged to be possibly related to budesonide, 10 were respiratory tract infection, 1 mild oral candidiasis and 1 osteoporosis. Of the 10 infectious episodes, 6 patients presented with mild forms of COVID-19. The infections were judged to be unrelated to budesonide treatment, but due to the ongoing COVID-19 pandemic. During these events, 3 patients presented gross hematuria that remitted with the resolution of the infection. However, the infections did not lead to hospitalizations. The case with osteoporosis was thought to be related to the post-menopausal period and was not associated with any fractures.

**Table 4:** Adverse events.

Adverse event	N (%)
Gastro-intestinal symptoms	3 (9.3%)
Gastrointestinal bleeding	0 (0%)
Respiratory tract infection	10 (31.2%)
COVID-19	6 (18.7%)
Oral candidiasis	1 (3.1%)
Osteoporosis	1 (3.1%)
New-onset diabetes mellitus	0 (0%)
Osteonecrosis	0 (0%)
Worsening of hypertension	0 (0%)

**Figure 6:**
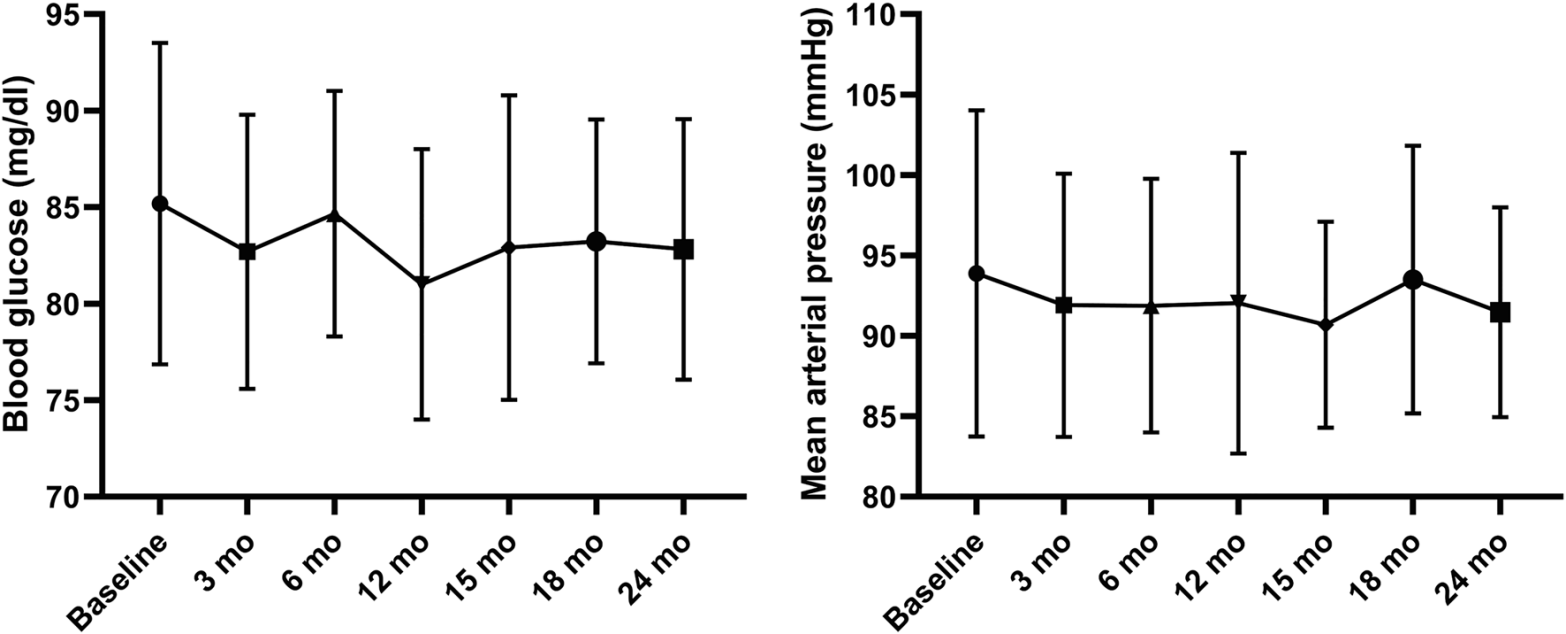
Blood glucose and mean arterial pressure evolution.

In addition, there were no patients with new-onset diabetes mellitus, gastro-intestinal bleeding, osteonecrosis or worsening of preexisting arterial hypertension. The blood glucose levels and mean arterial pressures remained stable throughout the study period (Fig. 6). In addition, none of the patients needed any new antihypertensive agents.

## Discussion

In this study, we have identified a significant impact of budesonide (Budenofalk) treatment in terms of reducing proteinuria (from a mean 1.89 g/d at baseline to 0.5 g/d at 36 months, corresponding to a mean percentage reduction of -68.1%) and preserving the renal function in patients with IgAN at high risk of progression. Overall, budesonide treatment was well tolerated with mild-moderate adverse events that did not lead to treatment discontinuation and no steroid-related side effects.

IgAN is associated with poor outcomes, with a kidney survival in adult patients of approximately 11 years and a mean age at kidney failure of 49 years^3^. Moreover, the patients previously considered to be at low risk of progression based on the KDIGO defined threshold of proteinuria below 1 g/d, still have a significant risk of kidney failure on long-term follow-up^4^. As such, it was recently shown that even patients with UPCR below 0.44 g/g reach ESRD within 10 years of diagnosis. Moreover, an eGFR decline as low as 1 ml/min/y would lead to progression to ESRD in 40% of patients diagnosed before 50 years of age, while an eGFR decline < 1 ml/min/y should be maintained to prevent kidney failure^3^. It became clear that even in these scenarios IgAN cannot be considered a benign disease^3^. Nonetheless, with current therapies the eGFR decline remains unacceptably high: − 1.4 ml/min/y (immunosuppression arm of the STOP-IgAN trial), − 2.5 ml/min/y (methylprednisolone arm of the TESTING trial) and − 3.5 ml/min/y (dapagliflozin arm of DAPA-CKD trial)^6, 9, 23^. Whether aiming for an earlier diagnosis of IgAN, use a combination of these agents or treat IgAN at lower levels of proteinuria would lead to an increase efficacy of current therapies remains to be established^3, 10^.

During the past decades significant progress had been made in elucidating the pathogenesis of IgAN paving the way for the development of targeted therapies^11–13, 24, 25^. Targeting the earliest pathogenic event in IgAN, the production of Gd-IgA1 molecules by the B-cells from the gut-associated lymphoid tissue, might provide a more sustained impact on long-term eGFR in such patients^10^. The known association between inflammatory bowel disorders (IBD) and IgAN led us to reconsider the potential utility of the locally acting glucocorticoid budesonide (Budenofalk) in the treatment of IgAN^26–29^. Budenofalk is a gastro-resistant, pH-modified formulation of budesonide with a maximum release of active compound in the distal ileum and proximal colon, that was approved for the treatment of mild-moderate Crohn’s disease^17, 18^. In a previous retrospective study, we have shown that a 24-months treatment regimen with budesonide (9 mg/d for 12 months, with dose tapering to 3 mg/d for another 12 months) led to a median reduction in proteinuria by 45% (IQR: − 79 to − 22%) and a stabilization of eGFR with a mean decline of − 0.22 ml/min/1.73m^2^ over the entire 24 months of follow-up^19^. This has led to the development of the BUDIGAN study, a prospective, single-arm study, evaluating the same budesonide treatment regimen over 24 months with an additional 12 months of post-treatment follow-up. Similar to our previous report, we confirmed that budesonide treatment was associated with a mean reduction of proteinuria of − 49.7% (95% CI − 70.1 to − 29.4) at 24 months and an eGFR change of + 0.83 ml/min/y (95% CI − 0.54 to 4.46) throughout the follow-up period. Proteinuria reduction has been accepted as a surrogate end point to assess a treatment’s effect on long-term renal function in IgAN^5, 30, 31^. Accordingly, a 40 and 60% reduction in time-averaged proteinuria led to a reduction in the rate of eGFR loss from − 5.65 ml/min/y to − 4.58 ml/min/y and–3.73 ml/min/y, respectively^3^. This magnitude of proteinuria decrease was associated with a 40–50% reduction in the risk for ESRD^3^. In our study, the eGFR change/y was substantially better than previous studies evaluating either systemic steroid therapy or SGLT2 inhibitors, possibly suggesting a better control of the underlying pathogenic process. Moreover, proteinuria continued to decrease even after budesonide treatment was stopped, suggesting a “legacy effect”^1^. Contrary, the eGFR tended to increase during the treatment period with a slight decline to baseline values in the post-treatment follow-up. This evolution of the renal function might be explained by several factors. First, the eGFR improvement during treatment might reflect a better control of the disease process with an attenuation of the effect following the treatment cessation. This would suggest that in IgAN a treatment for a limited period of time might be insufficient to significantly alter the long-term renal outcome of this chronic autoimmune condition and a longer treatment duration might be necessary. This is supported by the observation that the effect on proteinuria ceased by 3 years with the use of a 6–8-month regimen of systemic steroids^9^. However, in our study proteinuria continued to decrease even after treatment cessation suggesting that other factors might explain the eGFR evolution. Although the therapeutic effect of budesonide could have been supported by a parallel decline in the level of Gd-IgA1, this was not monitored in our study^32–34^. Second, a systemic effect of the locally acting budesonide might be responsible for the eGFR increase during treatment. Despite that a sarcopenic effect or glomerular hyperfiltration have been described with the use of systemic steroids, such an effect is unlikely with the locally acting budesonide^9^. A glomerular hyperfiltration effect should have been associated with an increase in proteinuria or worsening of preexisting hypertension. However, the current doses used in our study, that were derived from IBD-treatment regimens, are associated with less the 10% of active compound reaching the systemic circulation^17, 18^. In addition, we did not identify worsening of hypertension among the adverse events suggesting that indeed the systemic effect of budesonide is minimal.

Further supporting the efficacy of budesonide in the treatment of IgAN is that reduction of proteinuria and eGFR stabilization was paralleled by a significant decline in hematuria, with over 90% of patients achieving a remission of hematuria by the end of follow-up. Although hematuria was not included as an endpoint in the recent trials evaluating either systemic (TESTING trial) or locally-acting steroids (NEFIGAN and NefIgArd trials), hematuria might be a surrogate marker of intraglomerular inflammation^9, 15, 16^. In addition, budesonide is presumed to selectively inhibit the mucosal immune system, decrease the production of B-cell activating factors and ultimately lower circulating Gd-IgA1-containing immune complexes^35^. While treatment with targeted-release formulation (TRF)-budesonide led to a significant decrease in circulating levels of Gd-IgA1-containing immune complexes in the NEFIGAN trial, in our study we could not assess the impact of treatment with this formulation of budesonide on serum levels of Gd-IgA1-containing immune complexes^36^. Whether hematuria improvement is associated with a similar impact on the autoimmune process with budesonide treatment in IgAN remains to be proven. While appealing, a comparison between TRF-budesonide and other enteric-coated cannot be currently made due to the absence of a definitive proof on the postulated mechanism of action and the different designs of these studies^35^.

The benefit of budesonide in patients with the most severe forms of IgAN remains unclear. Although our study included 6 patients with crescents (in less than 30% of the examined glomeruli), the impact of budesonide on renal outcome in those with C2 lesion is heterogeneous as one patient still showed a rapid loss of eGFR. In these patients, an initial approach with systemic steroids (± other immunosuppressive agents) may be regarded as more appropriate to mitigate the severe intraglomerular inflammation, followed by a maintenance therapy with budesonide to control the autoimmune process^36^.

In terms of the infections risk, 31.2% of the study cohort had a respiratory tract infection throughout the follow-up, with 18.7% of patients having a COVID-19. However, by comparison to systemic steroids, these infectious events were mild-moderate and did not lead to hospitalization or treatment discontinuation. Moreover, the study was conducted through the COVID-19 pandemic and the SARS-CoV-2 infections were considered to be related to the ongoing pandemic and not to a systemic immunosuppressive effect of budesonide.

Our study has several limitations that need to be acknowledged. First, this is a single-center, open label study with no control arm, that enrolled a limited number of patients (n = 32). However, this should be regarded more like a hypothesis generating study and these results should be replicated in a randomized-control fashion versus, other formulations of budesonide or systemic steroids. Second, we did not measure the levels of Gd-IgA1 which would have supported our hypothesis of a disease-modifying effect of budesonide. Third, 15.6% of the study cohort showed a rapid progression as defined by the KDIGO guideline (eGFR decline over 5 ml/min/y)^37^, with one patient reaching an eGFR below 10 ml/min/1.73m^2^ by the end of follow-up period. This might be related to the inclusion of patients with an eGFR below 30 ml/min/1.73m^2^ (15.6%), which suggests limited efficacy of budesonide treatment in this subgroup of patients. In addition, the dosage of budesonide was derived from the treatment of mild-moderate active Crohn’s disease and whether higher doses are needed to better modulate the distant glomerular inflammation remains to be answered. Fourth, although we adequately explored for all adverse events during the study period, the systematic measurement of bone density was not undertaken in all patients, but only in selected cases (e.g. older females in the postmenopausal period). This was mainly due to the absence of evidence for an increased risk of osteoporosis associated with oral budesonide use in patients with IBD, and the relatively young age of the study cohort with no particular risk factors for osteoporosis^38^.

In conclusion, budesonide (Budenofalk) was effective in the treatment of patients with IgAN at high-risk of progression in terms of reducing proteinuria and preserving renal function over 36 months of therapy.

## Data Availability

All data generated or analyzed during this study are included in this published article.
